# Correction: Synthesis and anticancer evaluation of [d-Ala]-nocardiotide A

**DOI:** 10.1039/d4ra90013h

**Published:** 2024-03-04

**Authors:** Rani Maharani, Muhamad Imam Muhajir, Jelang Muhammad Dirgantara, Ari Hardianto, Tri Mayanti, Desi Harneti, Nurlelasari, Kindi Farabi, Ace Tatang Hidayat, Unang Supratman, Teruna Siahaan

**Affiliations:** a Department of Chemistry, Faculty of Mathematics and Natural Sciences, Universitas Padjadjaran Jatinangor West Java Indonesia r.maharani@unpad.ac.id; b Central Laboratory, Universitas Padjadjaran Jalan Raya Bandung-Sumedang KM 21, Jatinangor 45363 West Java Indonesia; c Centre of Natural Products and Synthesis Studies, Faculty of Mathematics and Natural Sciences, Universitas Padjadjaran Jalan Raya Bandung-Sumedang KM 21, Jatinangor 45363 West Java Indonesia; d Department of Chemistry, Graduate School of Science, Osaka University Toyonaka Osaka 560-0043 Japan; e Department of Pharmaceutical Chemistry, School of Pharmacy, The University of Kansas 2095 Constant Avenue Lawrence Kansas 66047 USA siahaan@ku.edu

## Abstract

Correction for ‘Synthesis and anticancer evaluation of [d-Ala]-nocardiotide A’ by Rani Maharani *et al.*, *RSC Adv.*, 2024, **14**, 4097–4104, https://doi.org/10.1039/D4RA00025K.

The authors regret that there was an error in the presentation of the units in Fig. 5 in the original article. The correct version of Fig. 5 is presented below.

**Fig. 5:**
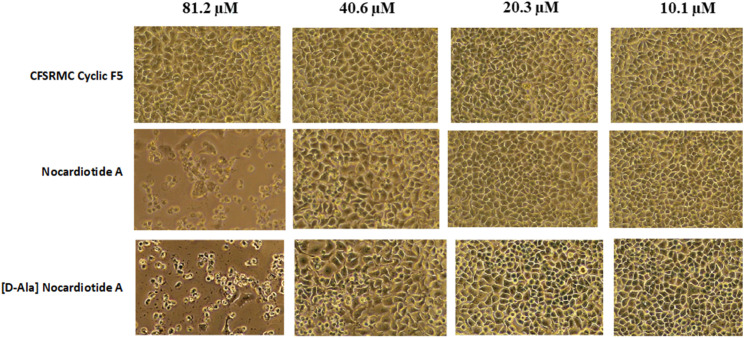
The effect of the concentrations of nocardiotide A, [d-Ala]-nocardiotide A, and CFSRMC cyclic F5 on the morphology of cervical cancer HeLa cells. Both nocardiotide A and [d-Ala]-nocardiotide A completely disrupted the monolayer integrity of HeLa cells at a concentration of 62.50 μg mL^−1^, while CFSRMC cyclic F5 as a negative control did not disrupt the monolayer integrity of HeLa cells.

The Royal Society of Chemistry apologises for these errors and any consequent inconvenience to authors and readers.

## Supplementary Material

